# Risk factors and nomogram predictive model of severe postoperative complications in elderly patients with intertrochanteric fractures

**DOI:** 10.12669/pjms.40.7.9242

**Published:** 2024-08

**Authors:** Ping Xu, Yanqiu Xu

**Affiliations:** 1 Ping Xu, Department of Orthopedics, Suzhou Hospital of Integrated Traditional Chinese and Western Medicine, 39 Xiashatang, Suzhou, Jiangsu Province 215000, P.R. China; 2 Yanqiu Xu, Department of Surgery, Suzhou Hospital of Integrated Traditional Chinese and Western Medicine, 39 Xiashatang, Suzhou, Jiangsu Province 215000, P.R. China

**Keywords:** Elderly, Intertrochanteric fracture, Severe postoperative complications, Risk factors, Nomogram

## Abstract

**Objective:**

To analyze risk factors of severe postoperative complications in elderly patients with intertrochanteric fractures (ITF), and to construct a predictive model.

**Methods:**

The medical records of 316 elderly patients with ITF who underwent surgical treatment in Suzhou Hospital of Integrated Traditional Chinese and Western Medicine from January 2020 to December 2022 were retrospectively analyzed. Univariate and multivariate logistic regression analyses were performed to identify risk factors of severe postoperative complications. A nomogram prediction model was constructed using the RMS package of R4.1.2 software. Accuracy and stability of the model was assessed using the receiver operating characteristic (ROC) curve, Hosmer-Lemeshow goodness-of-fit test, and decision curve analysis.

**Results:**

Age, American Society of Anesthesiologists (ASA) grading, combined medical diseases, preoperative bedridden condition, frailty, and preoperative albumin levels were all risk factors for severe postoperative complications in ITF patients were noted. These factors were then used to build a risk prediction model that had an area under the ROC curve (AUC) of 0.899 (95% confidence interval (*CI)*: 0.846-0.951). The internal validation results of the Bootstrap method showed that the C-index value of the model was 0.899, and the calibration curve had a good fit with the ideal curve.

**Conclusions:**

Age, ASA grading, combined medical diseases, preoperative bedridden condition, frailty, and preoperative albumin levels were independent risk factors for severe postoperative complications in elderly ITF patients. The constructed prediction model based on the above risk factors has a high predictive value.

## INTRODUCTION

Intertrochanteric fracture (ITF) is common in the elderly patient that mainly results from falling from a standing position and the rate of ITF has been increasing in recent years.^1^ At present, the treatment of ITF includes conservative management and surgical treatment.^2^,^3^ Conservative management requires long-term bed rest, which may increase the risk of pneumoconiosis and stress injury that can interfere with fracture healing and functional rehabilitation.^3^ Therefore, most ITF patients without obvious contraindication will be treated with surgery.^4^

The combination of surgical approach and early guidance of functional exercise can effectively promote functional recovery and reduce mortality in patients with ITP.^3^,^4^ However, elderly patients with ITF often present with multiple comorbidities. Additionally, due to the general frailty of this population, there is a risk of multiple complications after surgery.^4^,^5^ Severe postoperative complications such as venous thrombosis, pulmonary embolism and respiratory failure not only affect the rehabilitation, but may also lead to patient’s death.^3^-^5^ Therefore, the prevention and management of severe complications after ITF surgery has been a key concern in clinical practice. The development and implementation of such preventive and management measures need to be based on a clear understanding of relevant risk factors.^4^,^6^ A large number of clinical studies have shown that age, comorbidities, and nutritional status are all important factors affecting the outcomes of ITF surgery and the incidence of postoperative complications.^7^,^8^ Despite this, there are few studies constructing a nomogram model for predicting severe postoperative complications in elderly patients with ITF.

The aim of this study was to conduct a comprehensive analysis of the risk factors of severe postoperative complications of ITF, and to construct a predictive model that may accurately identify high-risk populations in the early stage.

## METHODS

Medical records of 316 patients (162 males and 154 females) with ITF who underwent surgical treatment in Suzhou Hospital of Integrated Traditional Chinese and Western Medicine from January 2020 to December 2022 were retrospectively analyzed.

### Ethical Approval

This clinical research conforms to the Declaration of Helsinki and fulfills relevant ethical requirements. This study was approved by the medical Ethics Committee of Suzhou Hospital of Integrated Traditional Chinese and Western Medicine for research (No. 2023-004, Date: 2023 August).

### Inclusion criteria:

- ITF caused by low-energy injury.^9^
- Age ≥ 60 years.
- All patients underwent proximal femoral anti rotation intramedullary nail fixation surgery, and the surgery was performed by the same group of doctors.
- The anesthesia method was combined lumbar epidural anesthesia.
- Complete medical records available.

### Exclusion criteria:

- Fractures caused by high-energy injuries such as high-altitude falls and traffic accidents.
- Additional injuries or fractures in other parts.
- Patients with pathological fractures.
- Patients with multiple fractures.
- Presence of limb dysfunction before the injury.

Severe postoperative complications included: venous thrombosis, pulmonary embolism, respiratory failure, deep infection at the operation site, septic shock, myocardial infarction, and acute renal failure. All complications of the patients were recorded at discharge.

The following patient information was collected: age, gender, body mass index (BMI), fracture stability, fracture to operative time, ASA grading, combined medical diseases, preoperative bedridden condition, asthenic condition, preoperative albumin level, and preoperative C-reactive protein (CRP) level. The stability of fractures was assessed based on the Evans Jensen classification standard, where Type I and II are stable fractures, while Type-III and IV are unstable fractures.^10^ Asthenic condition was evaluated using the Fatigue, Resistance, Ambulation, Illnesses, & Loss of Weight (FRAIL) Scale, which includes five questions: fatigue, low resistance, low mobility, decreased body mass, and multiple illnesses.^11^ Each question has a score of one, with a total score of 0 indicating no weakness, 1-2 indicating early weakness, and ≥ three indicating weakness. The postoperative complications of the patients were recorded at the discharge that was considered an observation endpoint.

### Statistical analysis

SPSS25.0 and R software version 4.0.0 were used for statistical analysis. Shapiro-Wilk test was used to evaluate the normality of data distribution. Data of normal distribution were expressed as mean ± standard deviation, and the inter group comparison was performed by independent sample *t* test. Data of non-normal distribution were expressed by median and interquartile interval, and Mann Whitney *U* test was used for inter group comparison. Counting data were expressed by n (%), and Chi-squared test was used for comparison between groups. Univariate and multivariate logistic regression model was used to analyze the risk factors of complications. A nomogram prediction model was constructed based on the identified independent risk factors. Receiver operating characteristic (ROC) curve and calibration curve were used to evaluate the discrimination and consistency of the model, respectively. *P*<0.05 was considered statistically significant.

## RESULTS

**Fig.1:**
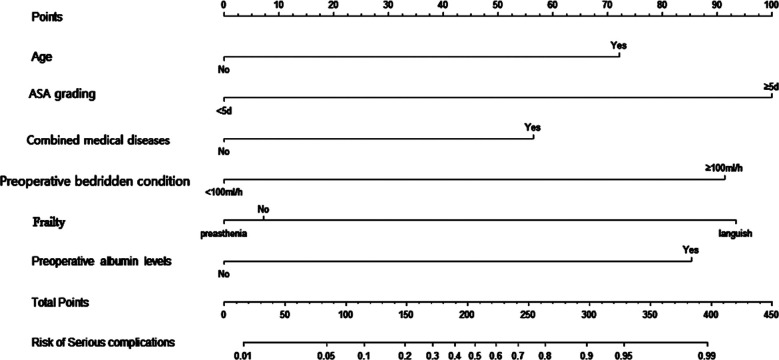
Nomogram model of severe postoperative complications in elderly of intertrochanteric fractures.

A total of 316 patients were included in this study. Age of the patients ranged from 60 to 83 years, with a mean age of 66.5 ± 3.8 years. There were 52 patients who had severe complications after the surgery, including 15 cases of venous thrombosis (28.8%), five cases of pulmonary embolism (9.6%), six cases of respiratory failure (11.5%), eight cases of deep infection at the operation site (15.4%), five cases of septic shock (9.6%), six cases of myocardial infarction (11.5%), and seven cases of acute renal failure (13.5%). There were significant differences in age, BMI, fracture to operative time, ASA grading, incidence of two or more internal medical diseases, preoperative bedridden condition rate, frailty, preoperative albumin levels, and preoperative CRP between patients with and without complications (*P*<0.05). There was no statistically significant difference in gender and fracture stability (P>0.05). Table-I. As shown in Table-II, age, ASA grade, combined medical diseases, preoperative bedridden condition, weakness, and preoperative albumin levels were risk factors for severe postoperative complications of ITF. Based on the results of multivariate logistic regression analysis, the data was imported into R software to construct a nomogram risk prediction model. Fig.1

**Table-I:** Univariate analysis of postoperative inflammatory complications of intertrochanteric fracture in elderly patients.

	Control group (n=264)	Observation group (n=52)	X^2^/t/Z	P
Age(years)	66(63,68)	70(67,73)	-6.926	0.000
Gender [n (%)]			0.253	0.615
Male	137(51.89)	25(48.08)		
Female	127(48.11)	27(51.92)		
BMI (kg/m^2^)	25.99(24.55,27.49)	26.82(25.13,29.53)	-2.557	0.011
Fracture stability [n (%)]			0.822	0.365
Stable	208(78.79)	38(73.08)		
Unstable	56(21.21)	14(26.92)		
Fracture to operative time (h)	43(40,47)	47(38.5,53)	-2.028	0.043
ASA grading [n (%)]			44.575	0.000
Class I	192(72.73)	18(34.62)		
Class II	65(24.62)	22(42.31)		
Class III	7(2.65)	12(23.08)		
Combined medical diseases [n (%)]			10.696	0.001
<2	227(85.98)	35(67.31)		
≥2	37(14.02)	17(32.69)		
Preoperative bedridden condition [n (%)]			37.651	0.000
No	260(98.48)	40(76.92)		
Yes	4(1.52)	12(23.08)		
Frailty [n (%)]			63.212	0.000
Non-frail	76(28.79)	6(11.54)		
Pre-frail	157(59.47)	15(28.85)		
Frail	31(11.74)	31(59.62)		
Preoperative albumin levels (g/L)	28.62±4.49	34.53±5.55	-8.330	0.000
Preoperative CRP levels (mg/L)	44.4(40.55,51.6)	48.65(44.75,55.75)	-3.173	0.002

**Table-II:** Multivariate logistic regression analysis results.

	B	S.E.	Wald	P	OR	95%CI
Age	1.887	0.662	8.118	0.004	6.598	1.802~24.160
BMI	0.822	0.433	3.606	0.058	2.276	0.974~5.3190
Fracture to operative time	0.632	0.445	2.014	0.156	1.882	0.786~4.506
ASA grading	2.019	0.607	11.052	0.001	7.532	2.291~24.771
Combined medical diseases	1.290	0.483	7.123	0.008	3.632	1.409~9.366
Preoperative bedridden condition	2.484	0.800	9.629	0.002	11.986	2.497~57.543
Frailty	1.398	0.347	16.228	0.000	4.049	2.050~7.994
Preoperative albumin levels	2.063	0.502	16.875	0.000	7.866	2.940~21.045
Preoperative CRP levels	0.350	0.620	0.318	0.573	1.418	0.421~4.783
Constant	-16.966	2.446	48.103	0.000	0.000	

**Fig.2:**
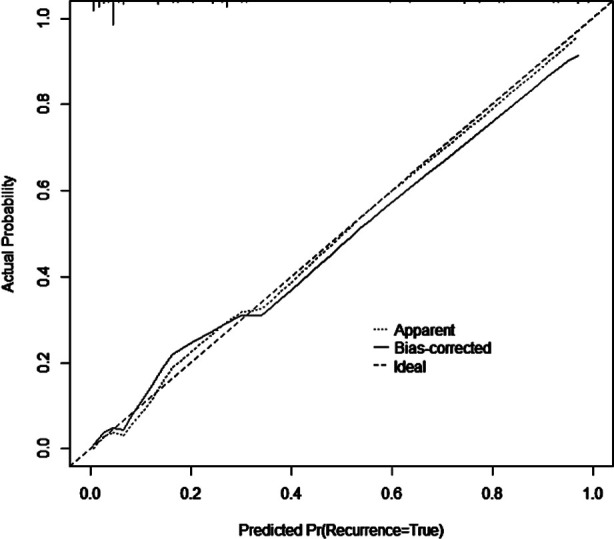
Calibration curve of the nomogram model.

**Fig.3:**
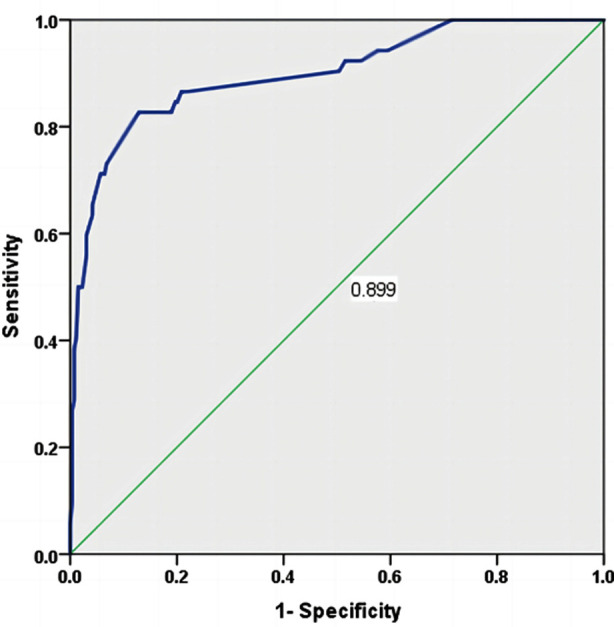
Analysis of Receiver operating characteristic of nomogram model.

The internal validation of the model was conducted using Bootstrap self-sampling method. After repeated sampling (1000 times), the consistency index (C-index) calculated was 0.899, and the calibration curve fitted the ideal curve well, indicating a high accuracy of the model. Fig.2 The ROC of the prediction model was drawn in R software, and had an AUC of 0.899 (95% CI: 0.846-0.951), indicating that it had certain predictive value for severe postoperative complications. When the optimal cut off value was selected, the sensitivity and specificity were 82.9% and 87.1%, respectively, indicating that the predictive model has a good effect. Fig.3

## DISCUSSION

The results of our study indicated that age, ASA grading, concomitant medical disease, preoperative bedridden condition, frailty, and preoperative albumin levels are all risk factors for severe postoperative complications in elderly patients with ITF. Our results showed that the incidence of severe postoperative complications in elderly patients with ITF was 16.5% (52/316). These results are consistent with previous studies that have also shown that the incidence of severe postoperative complications in elderly patients with ITF is 12% -54%.^6^,^12^,^13^ The occurrence of complications may be related to the living environment, patient’s tolerance to symptoms, definition of complications, and different implementation methods of surgical treatment.^14^,^15^ When choosing to treat elderly patients with ITF through surgery, it is necessary to continuously strengthen perioperative management, develop and implement a series of measures to avoid or reduce the risk of severe postoperative complications, and effectively reduce their incidence to further improve the prognosis.^16^,^17^

Age, ASA grading, comorbidities, preoperative bedridden condition, frailty, and preoperative albumin levels were independent risk factors of severe postoperative complications in our cohort of patients. The study by Vajapey *et al*^18^ showed that as age increases, organ function declines in elderly patients with ITF, and the impact of chronic diseases becomes more severe. Additionally, sensitivity to anesthesia and surgical trauma is higher in elderly population, and postoperative bed rest time is longer, which can lead to imbalance in the body’s homeostasis, leading to severe complications.

ASA grading is a common surgical risk assessment indicator. A study of Cammann *et al*^19^ found a positive correlation between ASA grading and postoperative risk in ITF patients, in agreement with our results. High ASA grading indicates that patients have poorer tolerance to anesthesia and surgery and may have a higher risk of severe complications after the surgery.

Comorbidities also significantly impacted the prognosis of elderly patients with ITF in our study. Patients with internal diseases such as cardiovascular, cerebrovascular, and respiratory systems have poorer physiological functions and are more prone to postoperative complications.^20^ The study by Chu *et al*^21^ showed that the combination of two or more internal medical diseases is an independent risk factor for postoperative mortality in elderly patients with ITF, further confirming the conclusions of our study.

Preoperative bedridden patients lack activity and have poor physiological and compensatory abilities that can further prolong the postoperative bedridden and recovery time, leading to severe postoperative complications.^22^ However, a study by Forssten *et al*^23^ reported that preoperative bedridden did not impact the rate of severe complications in patients after ITF. This discrepancy may be related to individual differences in the cohorts. Further studies are needed to verify the effect of being bedridden on the rate of postoperative complications in ITF patients.

Frailty is one of the indicators of physiological reserve function and a common predictive factor for postoperative complications in elderly surgical patients.^22^,^24^ The study by Son *et al*^25^ showed that frailty is an independent predictor of postoperative complications in elderly patients with hip fractures. Similarly, our study showed that elderly patients with frailty had poorer systemic compensatory ability, and therefore a higher likelihood of severe postoperative complications.

Serum albumin is a commonly used indicator of nutritional status. Bath *et al*^26^ showed that in elderly patients with ITF, serum albumin levels ≥ 35g/L was associated with a significantly reduced mortality rate. Elderly patients with ITF often have problems such as decreased muscle strength and decreased appetite, which can lead to malnutrition and increase the risks, associated with surgery.^2^,^27^

Current study identified the risk factors for severe postoperative complications in elderly patients with ITF. Moreover, the risk of severe postoperative complications was further calculated based on the nomogram model score. The prediction model that constructed based on the identified risk factors was shown to have a high predictive value, which is basically consistent with the conclusions by Shi et al.^28^ Since all indicators are relatively easy to obtain in clinical practice, appropriate preventive measures can be taken in the perioperative period. These include curative care of comorbidities, strengthening nutrition management, and rational design of anesthesia and surgery programs to minimize surgical trauma and reduce the incidence of severe postoperative complications to the greatest extent possible. Taken together, these measures may further improve patient prognosis.

### Limitations

This is a single center retrospective analysis, which may result in selection and information bias. Moreover, no long-term follow-up has been conducted and no additional possible risk factors have been included, indicating a certain degree of subjectivity and one-sidedness in the conclusion. The model constructed this time has a small sample size and was validated using internal validation methods. There is still room for further revision and adjustment of prospective data from large samples and multiple centers to better meet clinical needs.

## CONCLUSION

The independent risk factors for severe postoperative complications of elderly ITF include age, ASA grade, comorbidities with internal medical conditions, preoperative bedridden condition, weakness, and preoperative albumin levels. The prediction model constructed based on the above risk factors has high predictive value.

### Authors’ contributions:

**PX:** Conceived and designed the study.

**PX and YX:** Collected the data and performed the analysis.

**PX:** Was involved in the writing of the manuscript and is responsible for the integrity of the study.

All authors have read and approved the final manuscript.

## References

[1] Wang B, Liu Q, Liu Y, Jiang R (2019). Comparison of Proximal Femoral Nail Antirotation and Dynamic Hip Screw Internal Fixation on Serum Markers in Elderly Patients with Intertrochanteric Fractures. J Coll Physicians Surg Pak.

[2] Malafarina V, Reginster JY, Cabrerizo S, Bruyère O, Kanis JA, Martinez JA (2018). Nutritional Status and Nutritional Treatment Are Related to Outcomes and Mortality in Older Adults with Hip Fracture. Nutrients.

[3] Li X, Xu J (2022). Comparison of proximal femoral nail antirotation and total hip arthroplasty in the treatment of femoral intertrochanteric fracture. Pak J Med Sci.

[4] Ackermann L, Schwenk ES, Lev Y, Weitz H (2021). Update on medical management of acute hip fracture. Cleve Clin J Med.

[5] Yang YP, Dong LK (2019). Alleviation of Postoperative Delirium by Spinal Anesthesia in Elderly Patients with Hip Fracture. J Coll Physicians Surg Pak.

[6] Berggren M, Karlsson Å, Lindelöf N, Englund U, Olofsson B, Nordstrom P (2019). Effects of geriatric interdisciplinary home rehabilitation on complications and readmissions after hip fracture:a randomized controlled trial. Clin Rehabil.

[7] Chen Y, Han Y, Niu Z, Pu W, Tao R, Lei Y (2021). Is Decreased Local Bone Quality an Independent Risk Factor for Complications Following Fracture Fixation of Facial Bones. J Craniofac Surg.

[8] Istianah U, Nurjannah I, Magetsari R (2021). Post-discharge complications in postoperative patients with hip fracture. J Clin Orthop Trauma.

[9] American Academy of Orthopaedic Surgeons Hip Fractures in Older Adults.

[10] Pang B, Li F, Zhong C, Weng X, Xu H, Yang T (2022). Comparative reliability study on classification of femoral intertrochanteric fractures by using Tang and Japanese new typing systems based on 3-D CT and Evans-Jensen and AO/OTA-2018 classical typing systems based on X-ray. J Radiat Res Applied Sci.

[11] Morley JE, Malmstrom TK, Miller DK (2012). A simple frailty questionnaire (FRAIL) predicts outcomes in middle aged African Americans. J Nutr Health Aging.

[12] Tsuda Y, Yasunaga H, Horiguchi H, Fushimi K, Kawano H, Tanaka S (2016). Complications and Postoperative Mortality Rate After Surgery for Pathological Femur Fracture Related to Bone Metastasis:Analysis of a Nationwide Database. Ann Surg Oncol.

[13] Weng Y, Cai Y, Li Z, Guo B, Xu J (2023). Construction and validation of a model for predicting postoperative severe complications of intertrochanteric fracture in the elderly. Chin J Tissue Engi Res.

[14] Tahir M, Ahmed N, Samejo MQA, Jamali AR (2020). The Phenomenon of “Obesity Paradox”in Neck of Femur Fractures. Pak J Med Sci.

[15] Liu Q, Cao J, Kong J (2019). Effects of Percutaneous Kyphoplasty on Bone Metabolism and Oxidative Stress in Elderly Patients with Osteoporotic Spinal Fractures. J Coll Physicians Surg Pak.

[16] Bengoa F, Carrasco M, Amenábar PP, Schweitzer D, Botello E, Klaber I (2017). Perioperative care of older patients with hip fractures. Rev Med Chil.

[17] Shaikh SA, Hussain S, Ali Samejo MQ, Ahmed N, Jamali AR (2021). Osetosynthesis of Fractures neck femur with cannulated screws:Evaluation of risk factors for post-operative complications. J Pak Med Assoc.

[18] Vajapey SP, Li M (2021). Intramedullary Nailing of a Periprosthetic Intertrochanteric Fracture in the Setting of Prior Hip Resurfacing:A New Technique for Fracture Fixation. Techniques in Orthopaedics.

[19] Cammann S, Karabulut S, DeTemple DE, Oldhafer F, Kulik U, Schroeter A (2022). Antibiotic-Resistant Bacteria Colonizing the Bile Duct Are Associated with Increased Morbidity and Mortality after Resection of Extrahepatic Cholangiocarcinoma. Surg Infect.

[20] Yang Y, Wang T, Guo H, Sun Y, Cao J, Xu P (2022). Development and Validation of a Nomogram for Predicting Postoperative Delirium in Patients with Elderly Hip Fracture Based on Data Collected on Admission. Front Aging Neurosci.

[21] Chu Z, Wu Y, Dai X, Zhang C, He Q (2021). The risk factors of postoperative delirium in general anesthesia patients with hip fracture:Attention needed. Medicine (Baltimore).

[22] Forssten MP, Mohammad Ismail A, Ioannidis I, Wretenberg P, Borg T, Cao Y (2023). The mortality burden of frailty in hip fracture patients:a nationwide retrospective study of cause-specific mortality. Eur J Trauma Emerg Surg.

[23] Forssten MP, Mohammad Ismail A, Borg T, Ahl R, Wretenberg P, Cao Y (2021). Postoperative mortality in hip fracture patients stratified by the Revised Cardiac Risk Index:a Swedish nationwide retrospective cohort study. Trauma Surg Acute Care Open.

[24] Yan B, Sun W, Wang W, Wu J, Wang G, Dou Q (2022). Prognostic significance of frailty in older patients with hip fracture:a systematic review and meta-analysis. Int Orthop.

[25] Son YJ, Shim DK, Seo EK, Won MH (2021). Gender differences in the impact of frailty on 90-day hospital readmission in heart failure patients:a retrospective cohort study. Eur J Cardiovasc Nurs.

[26] Bath J, Smith JB, Woodard J, Kruse RL, Vogel TR (2021). Complex relationship between low albumin level and poor outcome after lower extremity procedures for peripheral artery disease. J Vasc Surg.

[27] Hart A, Sun Y, Titcomb TJ, Liu B, Smith JK, Correia MLG (2022). Association between preoperative serum albumin levels with risk of death and postoperative complications after bariatric surgery:a retrospective cohort study. Surg Obes Relat Dis.

[28] Shi H, Gao Y, Zhao W, Wang H, Wu X, Wang F (2023). Development of a prediction model for postoperative complications and economic burden analysis in older patients with hip fractures. Heliyon.

